# Discovery of 4-(2-(dimethylamino)ethoxy)benzohydrazide derivatives as prospective microtubule affinity regulating kinase 4 inhibitors†

**DOI:** 10.1039/d0ra00453g

**Published:** 2020-05-27

**Authors:** Nashrah Sharif Khan, Parvez Khan, Afreen Inam, Kamal Ahmad, Mohd. Yousuf, Asimul Islam, Sher Ali, Amir Azam, Mohammad Husain, Md. Imtaiyaz Hassan

**Affiliations:** Department of Biotechnology, Jamia Millia Islamia Jamia Nagar New Delhi 110025 India mhusain2@jmi.ac.in; Centre for Interdisciplinary Research in Basic Sciences, Jamia Millia Islamia Jamia Nagar New Delhi 110025 India mihassan@jmi.ac.in; Department of Chemistry, Jamia Millia Islamia Jamia Nagar New Delhi 110025 India aazam@jmi.ac.in

## Abstract

Microtubule affinity regulating kinase 4 (MARK4) is a Ser/Thr kinase, considered as a potential drug target for cancer, diabetes and neurodegenerative diseases. Due to its significant role in the development and progression of cancer, different in-house libraries of synthesized small molecules were screened to identify potential MARK4 inhibitors. A small library of hydrazone compounds showed a considerable binding affinity to MARK4. The selected compounds were further scrutinized using an enzyme inhibition assay and finally two hydrazone derivatives (H4 and H19) were selected that show excellent inhibition (nM range). These compounds have a strong binding affinity for MARK4 and moderate binding with human serum albumin. Anticancer studies were performed on MCF-7 and A549 cells, suggesting H4 and H19 selectively inhibit the growth of cancer cells. The IC_50_ value of compound H4 and H19 was found to be 27.39 μM and 34.37 μM for MCF-7 cells, while for A549 cells it was 45.24 μM and 61.50 μM, respectively. These compounds inhibited the colonogenic potential of cancer cells and induced apoptosis. Overall findings reflect that hydrazones/hydrazone derivatives could be exploited as potential lead molecules for developing effective anticancer therapies *via* targeting MARK4.

## Abstract 2

Inhibition studies of MARK4 with selected hydrazone derivatives.

## Graphical abstract

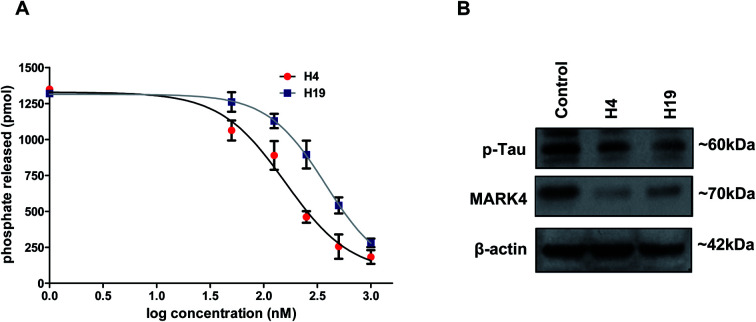

## 1 Introduction

Protein kinases catalyze the transfer of phosphate from ATP to Ser/Thr/Tyr side chains of specific target proteins to induce conformational changes and subsequent modulation in their activities.^1^ Protein phosphorylation is essential for the regulation of many fundamental cellular activities including metabolism, division, movement, apoptosis and survival. Any alteration in protein phosphorylation causes a change in cellular function and sometimes leads to severe diseases including cancer.^2^ Protein kinases activate various intra- and extracellular downstream signaling pathways associated with biological activities such as differentiation, growth and apoptosis.^3,4^ Thus, a large number of kinases are targeted for the design and development of effective therapeutic molecules to address associated diseases.^5–10^

Microtubules affinity regulated kinase 4 (MARK4), a Ser/Thr kinase belonging to the family of AMPK which is currently a well-established drug target for cancer, diabetes and neurodegenerative diseases.^11^ MARK4 regulates the dynamics of microtubule by its phosphorylation and thus plays a significant role in cell division, cell proliferation and cell cycle regulation.^12^ Recently, MARK4 has shown to be involved in the progression and migration of breast cancer cells by inhibiting Hippo signaling.^13^ Overexpression of MARK4 results in hyperphosphorylation of tau protein which in turn leading to Alzheimer's disease and other tauopathies.^14^ In addition, MARK4 induces adipogenesis in 3T3-L1 adipocytes by activating the JNK1 pathway.^15^ Overexpression of miR-515-5p inhibits cell progression and migration by down-regulation of MARK4 mRNA levels in breast and lung cancer.^16^ Similarly, MARK4 plays key role in energy metabolism and homeostasis.^17^

Phosphorylation of conserved threonine residue (Thr214) by Liver Kinase B1 (LKB1) and MARKK/TAO-1 (MARK Kinase/Thousands and One amino acids) in the activation loop activates MARK4, while phosphorylation in Ser218 residue inactivates.^18^ MARK4 is a primary regulator of Wnt signaling pathway and high expression is linked with Wnt-induced prostate cancer, providing as a significant target for the development of anti-cancer drugs.^19^ The inhibition of MARK4 suppresses the progression of glioma.^20^ Many potential natural and synthetic molecules have been identified as MARK4 inhibitors.^9,21–27^ These inhibitors decrease the growth and proliferation of different cancer cell types and indicate the importance of MARK4 inhibitors to improve the outcomes of associated cancers. All these studies established MARK4 as a potential druggable target for cancer and other disorders.^24,28–31^

This study was performed to identify potential scaffolds for the development of effective MARK4 inhibitors. In pursuit of this, we have screened our in-house library for the identification of effective inhibitors for MARK4. Based on a series of *in silico* and *in vitro* screening assays, two hydrazone molecules were selected which possess considerable inhibition and high binding affinities for MARK4 and decrease the growth and proliferation of cancer cells. We found that the treatment of these molecules inhibits cell migration and induces apoptosis in selected cancer cells. Our results show the potential of hydrazone derivatives for the development of anticancer molecules which may be further exploited for clinical management of anticancer therapy.

## 2 Material and methods

### 2.1. Materials

The Luria broth and Luria agar were taken from BD Difco™ Franklin Lakes, NJ. Human cell lines (MCF-7, A549 and HEK-293) used were procured from National Centre for Cell Sciences, Pune, India. Phospho-Tau, MARK4 (MA5-27002). actin monoclonal antibodies, MTT (3-[4,5-dimethylthiazol-2-yl]-2,5-iphenyltetrazolium bromide), antibiotic cocktail, fetal bovine serum, Dulbecco's modified eagle's media (DMEM) and propidium iodide (PI) were procured from Gibco-life technologies, Thermo Fischer Scientific (USA). All other reagents were of analytical grade purchased from local supplier.

### 2.2. Molecular docking

Molecular docking based analysis was performed to see the possible interactions between the test compounds and MARK4 using our published protocol.^24,32^ Crystal structure of MARK4 (PDB ID 5ES1)^33^ was used for docking using Maestro 10.5 program (Schrodinger Inc. USA) on a 64 bit operating system [windows 7 with an HP computer, Intel ® Core ™ i5-2400 CPU @ 2.40 GHz, 6 GB RAM]. The input file of MARK4 was first prepared using the protein-preparation wizard module and the active site was generated as grid box using Glide.^30^ Structure of ligand molecules was drawn as mol file using ChemDraw 12.0 software and their energy was minimized using LigPrep module of Maestro. All possible ionization states at pH 7.0 ± 2.0 were generated and minimized. Ligands prepared were subjected to dock into the active site of MARK4 in extra precision mode (XP) using Glide.^34^ Exhaustive enumeration of ligand torsions generates a collection of ligand conformations that are examined during the docking process. Glide XP employs an anchor-and-grow sampling approach for GlideScore and dock compounds at a faster rate. These docking modes provide an array of options in the balance of speed *vs.* accuracy for most of the situations. PyMOL and DS visualizer were used for visualization, evaluation and analysis of protein–ligand docked structures.^35,36^ To validate the docking protocol, the reported co-crystallized inhibitor of MARK4 (pyrazolopyrimidine inhibitor) was re-docked into the binding site and subsequently compared with the crystal data.^30^

### 2.3. Enzyme inhibition assay

MARK4 protein was expressed and purified by following previous protocols.^27,37^ The purity of the protein was further confirmed by a single band shown by SDS PAGE. ATPase assay was carried out to evaluate the enzyme activity of MARK4 in the presence of selected hydrazone derivatives. The activity of MARK4 was studied with the increasing concentration of each selected hydrazone derivative by following our published protocol.^24,38^

### 2.4. Fluorescence measurement

The binding affinity of selected hydrazone derivatives with human serum albumin (HSA) and MARK4 was carried out using fluorescence binding studies as described.^39^ The values of binding constant (*K*_a_) and a number of binding sites present in protein (*n*) were obtained by using the modified Stern–Volmer equation from the intercept and slope, respectively.

### 2.5. Cell proliferation assay

Human cell lines (HEK-293, A549 and MCF-7) were seeded and maintained in a medium supplemented with DMEM along with 10% heat-inactivated fetal bovine serum (Gibco) and 1% penicillin, streptomycin solution (Gibco), cultured in a 5% CO_2_ humidified incubator at 37 °C. Cell cultures were maintained and trypsinized regularly, not for more than 30 passages. MTT assay was carried out to see the effect of selected hydrazone compound treatment on the viability of non-cancerous (HEK-293) and cancerous cell lines (A549 and MCF-7) as per described method.^22,24^ In brief, 6000–7000 cells per well were seeded in a 96-well plate and after 24 h growth, the cells were treated with increasing concentrations (0–200 μM) of each hydrazone (final volume of 200 μL) for 48 h at 37 °C in a humidified chamber. After 48 h, MTT was added (20 μL solution from 5 mg mL^−1^ stock solution prepared in phosphate buffer saline, pH 7.4) to each well and plates were further incubated for 4–5 h at 37 °C in a CO_2_ incubator. The supernatant was aspirated and the formazan crystals were dissolved in 150 μL of DMSO. The absorbance (*A*) of final products was measured using multiplate ELISA reader (BioRad) at 570 nm. The percentage of cell viability was calculated and IC_50_ (50% inhibitory concentration) values of each hydrazone derivative were estimated (paclitaxel was taken as a positive control for cell proliferation studies).

### 2.6. Colony formation assay

The colony-forming assay was performed to evaluate the effect of selected compounds on cell colonization potential of cancer cells. Briefly, cells were plated at a density of 1000–2000 (A549/MCf-7) cells per well in a six-well cell culture plate. Cells were grown for at least 24 h in cell growth medium and then incubated with IC_50_ concentration of selected compounds for 10–12 days at 37 °C (in a humidified 5% CO_2_ incubator). Control cells were treated with an equal percentage of DMSO. After 10–12 days, the cell colonies were fixed with 100% methanol and staining was performed using 0.4% crystal violet in 25% methanol. The colonies obtained was counted, plotted and compared with the controls.

### 2.7. Trans-well cell migration assay

Trans-well migration assay was performed using a modified Boyden chamber containing cell culture inserts (8 μm pore size, polyethylene terephthalate track-etched membrane, BD Falcon #353093). Briefly, cells were serum-starved for 3–4 h, trypsinized and suspended in a serum-free medium. In the upper chamber of cell culture insert, an equal number of cells with the IC_50_ dose of selected compounds or DMSO (control) were plated and in the lower chamber of well plate, 10% FBS-DMEM (for chemotaxis movement) was added. The cells were allowed to migrate for 24 h in a humidified 5% CO_2_ incubator at 37 °C and the migrated cells (that invaded through membrane pores) onto the lower side of Trans-well cell culture insert were fixed with methanol and staining was performed using 0.4% crystal violet in 25% methanol. The remaining cells present on the upper side of the chamber were wiped off with the help of cell scrapper/tissue wipes. Finally, the migrated cells present on the lower surface of the cell culture insert were imaged using a light microscope and counted in 3–5 random fields.

### 2.8. Protein isolation and western blot

The compound treated cell line samples were lysed in RIPA cell lysis buffer (Thermo Fisher Scientific, USA) in an ice-cold condition using ultrasonication. Total protein was isolated and protein estimation was performed using Bicinchoninic Acid Assay (BCA-protein estimation kit). Nearly 30–40 μg of whole protein lysate was diluted with 6× Laemmli's buffer, boiled for 3–5 min and resolved using 10–12% SDS-polyacrylamide electrophoresis under reducing conditions. The resolved polypeptides were transferred to polyvinylidene fluoride (PVDF) membrane through blotting, peptide-specific primary antibodies were used for identification and horseradish peroxidase coupled conjugates were used for visualization using luminol as a chemiluminescent substrate.^40^

### 2.9. Apoptotic cell assay

Annexin-V staining was used to assess the apoptotic potential of the selected hydrazones as described.^22,41^ Briefly, cells were treated with IC_50_ concentration of selected hydrazones for 24 h and control cells were treated with DMSO/culture medium only. After 24 h incubation, approximately 2.0–2.5 × 10^6^ cells were collected by trypsinization and washed twice with PBS (5–10 mL). Finally, FITC labelled Annexin-V and PE labelled PI was used for staining the cells using the FITC-Annexin-V kit by following manufacture instructions (BD-Biosciences, USA). Nearly, 10 000 events were recorded using BD LSR II Flow Cytometry Analyzer and analyzed by FlowJo software.

## 3 Results and discussion

### 3.1. Screening of hydrazone derivatives

We have screened a small library of hydrazone derivatives that shows a significant binding affinity with the MARK4. The results of screening and docking are shown in the ESI Fig. S1–S4.† Docking results suggested that selected molecules significantly bind with active site residues of MARK4. Following the *in silico* screening analysis, the selected compounds were synthesized as per our previously reported method.^42^ The scheme of synthesis and NMR characterization data is provided in the supplementary information (for details see ESI†).

**Fig. 1:**
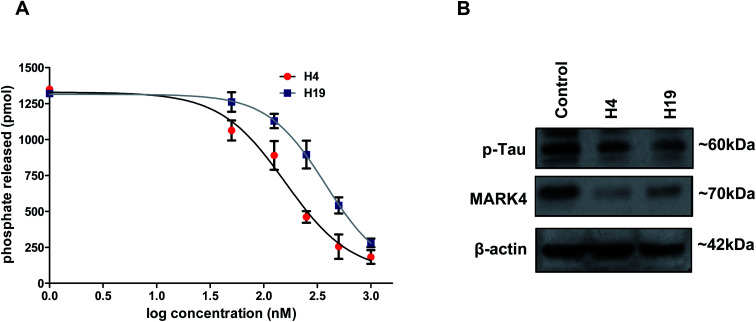
Inhibition studies of MARK4 with selected hydrazone derivatives. (A) Dose–response curve for % hydrolysis of ATP shows the effect of increasing concentrations of compound H4 and H19 on ATPase activity of MARK4. Released phosphate was quantified using standard phosphate curve. (B) Representative expression profile of MARK4 and p-tau in compound H4/H19 treated MCF-7 cells with respect to control. Immunoblotting studies were performed after stipulated time treatment of respective compound and showed that treatment of cells with compound H4/H19 decreases the protein levels of MARK4 and p-tau.

To further evaluate the inhibitory potential of selected compounds with MARK4, enzyme inhibition assay was performed (at a single dose of 10 μM). We found that at the selected dose of most of the molecule decreases the hydrolysis of ATP, but molecules H4 and H19 inhibit the activity with a great efficacy (IC_50_ < 1 μM). Thus, these two compounds were further selected and evaluated with MARK4 in a concentration-dependent manner. Interestingly, a progressive decrease in the activity of MARK4 was observed with the increasing concentration of molecules H4 and H19 (Fig. 1A). The percent inhibition of ATPase activity was plotted as a function of ligand concentration and used to estimate IC_50_ (concentration of 50% inhibition, Fig. 1A).

The IC_50_ concentration of compound H4 and H19 were calculated as 149.21 nm and 215.30 nm, respectively. The cell-free enzyme inhibition assay clearly suggests that the selected molecules (H4 and H19) inhibit MARK4 activity in the nano molar range. Next, we tried to see the inhibition potential of these molecules at the cellular level. Cells were treated with IC_50_ concentration of each molecule and processed for the expression studied of MARK4 and p-tau (substrate of MARK4). The results of immunoblotting showed that the treatment of compound H4 and H19 inhibited the expression of MARK4 (Fig. 1B). Interestingly, with respect to the expression of MARK4 the tau phosphorylation results also showed a similar pattern of phosphorylation. Phosphorylation of tau correspondingly decreases in the treated cells as compared to control (Fig. 1B). These results further confirmed that the selected hydrazone molecules significantly inhibited the kinase activity of MARK4.

### 3.2. Docking and interaction analysis

**Fig. 2:**
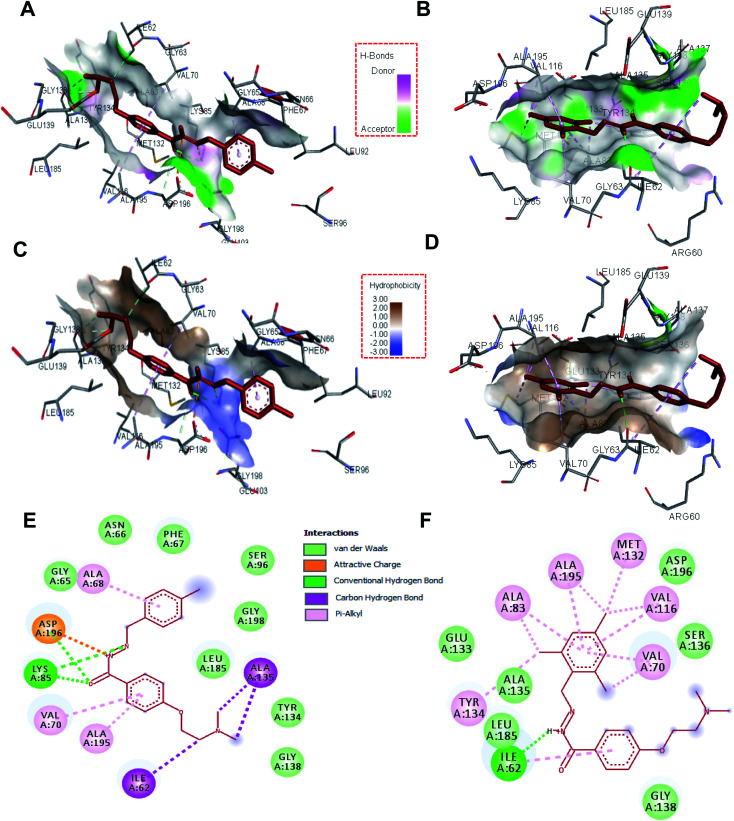
Molecular docking studies of compound H4 and H19 with MARK4: 3D focused view of MARK4 active site residues docked with (A) compound H4, (B) compound H19, shows the hydrogen-bond donor–acceptor residues of protein. Representation of MARK4 active site showing hydrophobicity of interacting residues with, (C) compound H4, (D) compound H19. 2D representation of residues involved in different interactions like van der Waals interactions, hydrogen bonding, charge or polar interactions with, (E) compound H4, (F) compound H19 (see inset for each type of interaction and respective colour).

To see the specific interacting residues and type of interactions between selected hydrazone derivatives, molecular docking analysis was carried out. Results suggest that these molecules bind to the active site of MARK4 with a high affinity. These two compounds mainly interact with Ile62, Gly65, Asn66, Phe67, Ala68, Val70, Lys85, Ser96, Tyr134, Ala135, Gly138, Leu185, Ala195, Asp196 and Gly198 of the binding cavity of MARK4 (Fig. 2). Analysis of the binding pattern suggests that the dimethylamino ethoxy moiety of compound H4 and H19 interacts with MARK4 in two different orientations. Dimethylamino ethoxy entity of H4 interacts with the Ile62 and Ala135 *via* carbon-hydrogen bonds, whereas in the case of H19 it extends towards the outer face of binding cavity. Another important pharmacophore of these molecules is hydrazone linkage which provides significant interactions to the residues of MARK4. It was found that hydrazone linkage of H4 forms two hydrogen-bonds with Lys85 and one with Asp196, similarly hydrazone moiety of H19 forms one hydrogen-bond with Ile62 (Fig. 2). To further validate our docking results and to get better insights into the binding modes, we have performed the docking studies of known inhibitor (co-crystallized ligand) of MARK4 and compared with these molecules. Results showed that these molecules occupy the same binding cavity of MARK4 as that of co-crystallized inhibitor (Fig. S5A and B†). It was found that compound H4 and co-crystallized inhibitor commonly binds with Ile62, Lys85, Tyr134, Ala135 and Asp196, whereas compound H19 shares Ile62, Lys85, Tyr134, Ala135 and Asp196 (Fig. S5C†). The comparative analysis of binding residues and type of interactions suggests that complex of MARK4 with H4 is almost mimicked the binding mode of MARK4-co-crystallized inhibitor and stabilized by many interactions.^30,33^ This might be a possible reason for a higher inhibition potential of H4 as compared to H19.

### 3.3. Fluorescence binding studies

**Fig. 3:**
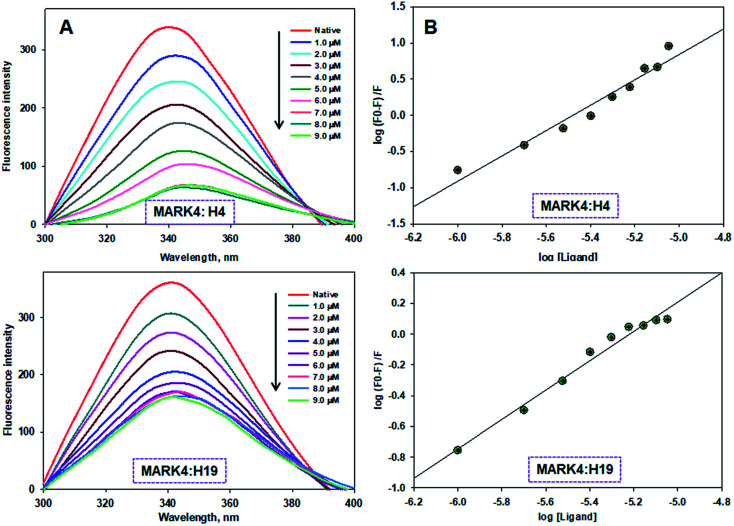
Binding of compound H4 and H19 with MARK4. (A) Fluorescence emission spectra of MARK4 (5 μM) with increasing concentrations of compound H4 and H19. (B) Stern–Volmer plot obtained from the fluorescence quenching data of MARK4 by compound H4 and H19, respectively. Protein sample was excited at 280 nm and emission for each titration was recorded in the range of 300–400 nm. The value of binding constant/number of binding sites was estimated using Stern–Volmer plot.

**Fig. 4:**
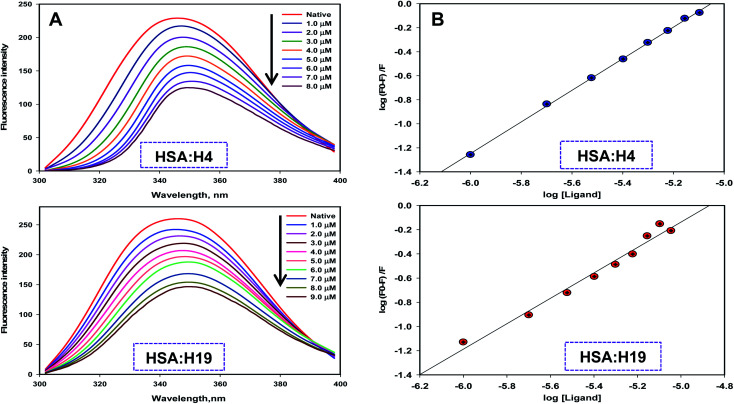
Binding of compound H4 and H19 with HSA. (A) Fluorescence emission spectra of HSA (5–10 μM) with increasing concentrations of compound H4 and H19. (B) Stern–Volmer plot obtained from the fluorescence quenching data of HSA by compound H4 and H19, respectively. Protein sample was excited at 280 nm and emission for each titration was recorded in the range of 300–400 nm. The value of binding constant/number of binding sites was estimated using Stern–Volmer plot.

To measure the actual binding affinity of selected ligands with MARK4 and HSA, fluorescence quenching studies were performed.^43,44^ Both MARK4 and HSA contain tryptophan residues, therefore it has been used as an intrinsic fluorophore to estimate the interaction of ligand. Protein was excited at 280 nm and the emission spectrum was recorded in 300–400 nm range. With increasing ligand concentrations (0–20 μM), the fluorescence intensity of MARK4 and HSA was decreases in a concentration-dependent manner (Fig. 3 and 4), indicating a considerable binding. Values of *K*_a_ and *n* were calculated from fluorescence emission spectra using the Stern–Volmer plot. The estimated values of *K*_a_ of H4 and H19 for MARK4 were 4.07 × 10^9^ M^−1^ and 9.58 × 10^7^ M^−1^, respectively. For HSA binding the values of *K*a H4 and H19 were calculated as 4.43 ×10^5^ M^−1^ and 1.30 ×10^5^ M^−1^, respectively. These results suggested that compound H4 and H19 show excellent binding affinity with MARK4; whereas, a moderate binding affinity is observed for HSA. Serum albumin is a major component of blood and helps in the transport of different molecules including drugs.^45^ The high binding affinity of these molecules with MARK4 advocates the formation of a stable complex with MARK4, whereas with HSA favors their blood transportable behaviour. Overall, the results of docking, fluorescence and enzyme activity are consistent and suggest an admirable binding affinity and inhibition potential for MARK4.

### 3.4. Cell proliferation studies

**Fig. 5:**
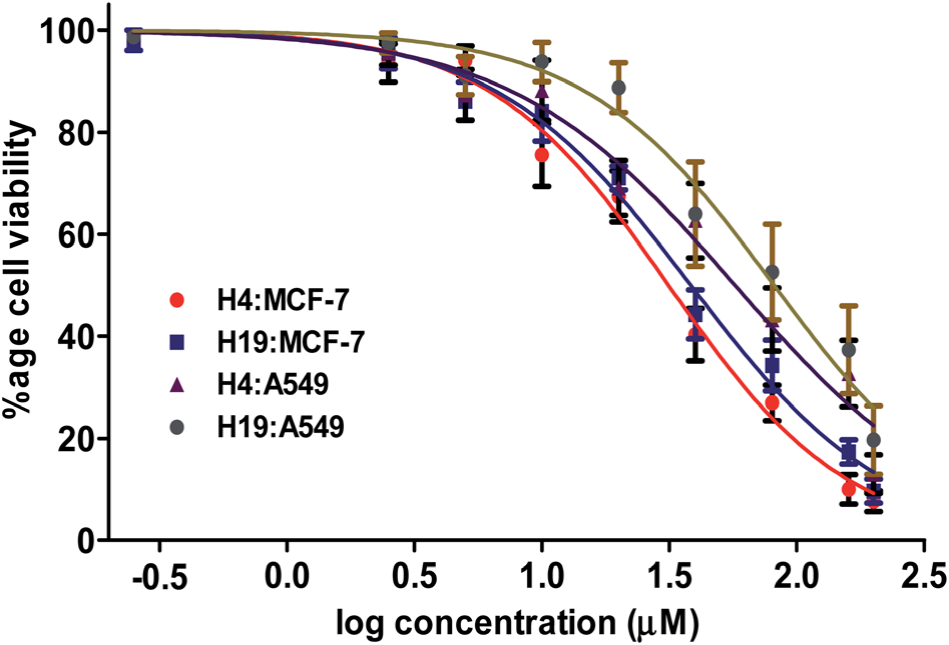
Compound H4 and H19 decreases the viability of MCF-7 and A549 cells. Cell viability studies of MCF-7 and A549 cells with increasing concentration of compound H4/H19 (0–200 μM) as quantified by MTT assay. Percent cell viability were estimated with respect to the untreated control cells. Each data point shown is the mean ± SD from *n* = 3.

Based on the results of enzyme inhibition and binding studies (*in vitro* and *in silico*), we selected compound H4 and H19 as promising inhibitors of MARK4. To further evaluate the pharmacological potential of these molecules, they were tested for cell based anticancer studies. MARK4 has a major role in the breast and lung cancer progression and metastasis, so anticancer properties of these molecules were evaluated on previously reported cell line models (MCF-7 and A549 cells).^16,46^ Treatment of H4 and H19 significantly inhibited the viability of selected cancer cells (Fig. 5). These compounds decrease the cancer cell proliferation and the estimated IC_50_ values for MCF-7 cells were 27.39 μM and 34.37 μM, respectively for H4 and H19. In case of A549 the IC_50_ values of H4 and H19 were 45.24 μM and 61.50 μM, respectively. In order to see the cytotoxicity of these compounds on normal cells, cell viability studies were performed on HEK293 cells. The cell viability results showed that the cytotoxicity dose of these compounds is higher for non-cancerous cells as compared to cancerous cells. These results suggest that at the IC_50_ concentration or active concentration compound H4 and H19 are less toxic to non-cancerous cells.

### 3.5. Colony formation and migration studies

**Fig. 6:**
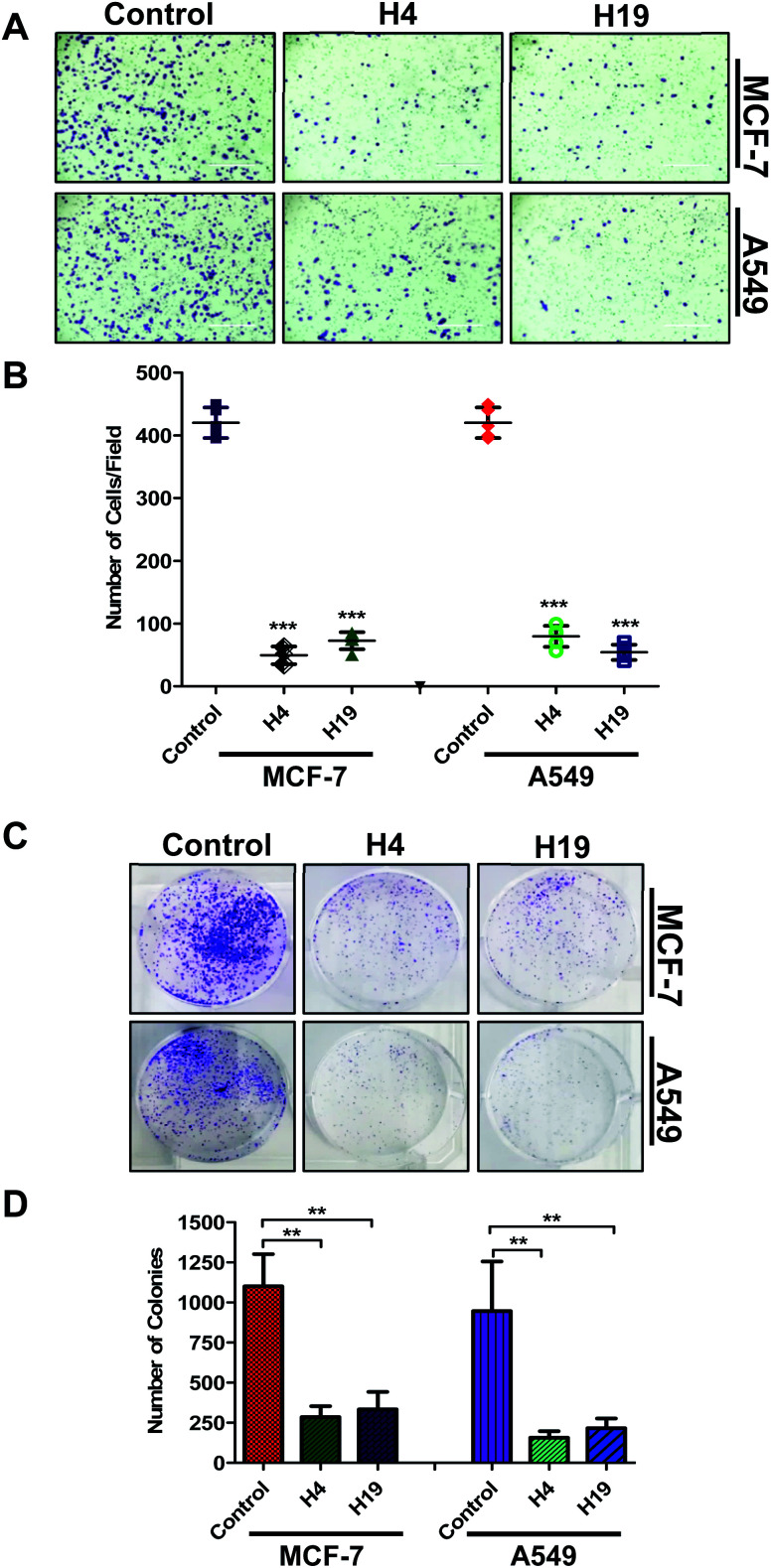
Treatment of compound H4 and H19 decreases cell migration and colonization potential of cancer cells. (A) Boyden chamber based Trans-well assay was used to know the effect of compound H4/H19 on the migration of MCF-7 and A549 cells. Migrated cells were fixed and stained with crystal violet. Representative images (20× original magnification) showing migrated cells. Scale bar represents 400 μ. (B) Bar graph represents the number of migrated cells under different treatments for triplicate measurements ± SD. (C) Colony formation assay used to study the effect of compound H4/H19 on colonization potential of MCF-7 and A549 cells. (D) Bar graph represents the number of colonies formed and compared with untreated control cells. ***p < 0.001 and **p < 0.01 as compared to control (untreated cells).

Colonization and migration are the characteristic features of cancer cells, thus anticancer molecules are expected to inhibit the colony formation and migration of cancer cells.^47^ With this assumption, the selected compounds were evaluated for colony formation and migration properties of MCF-7 and A549 cells. For this, the cells were treated with IC_50_ concentration and colony formation/migration potential was analysed. Interestingly, results showed that a lesser number of cells were migrated to the lower side of chamber in case of treated cells (Fig. 6A and B). In addition, the number of colonies were significantly decreased in H4 and H19 treated cells (Fig. 6C and D). Overall results indicate that molecule H4 and H19 inhibit the colonogenic as well as migration properties of MCF-7 and A549 cells.

### 3.6. Apoptotic studies

**Fig. 7:**
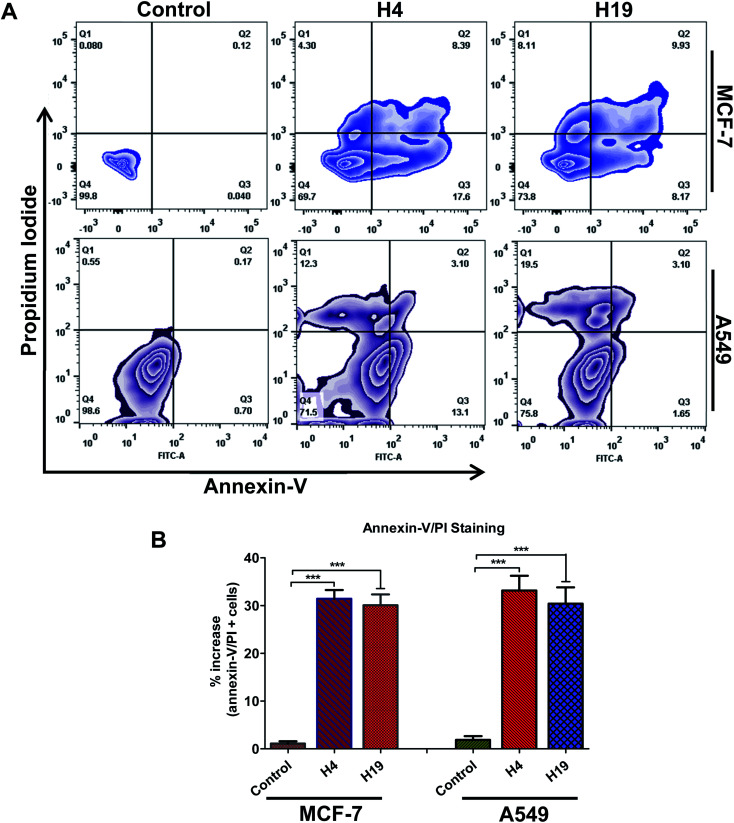
Compound H4 and H19 induces apoptosis in cancer cells. (A) Zebra plot showing the FITC-Annexin-V and PI stained cells for apoptosis analysis after the treatment of compound H4 and H19. (B) Bar graph represents the percentage of apoptotic cells stained with Annexin-V. Statistical analysis was performed using *t*-test for unpaired samples, ***p < 0.001, compared to the control (untreated cells).

Cancer cells support their growth and maintenance by evading the apoptotic pathways.^48^ MARK4 regulates the cancer cell growth and proliferation *via* modulating the apoptotic pathways.^12,15,46^ The treatment of MCF-7 and A549 cells with H4 and H19 inhibit the activity of MARK4, decreases cell proliferation and migration raises the evaluation of these MARK4 inhibitors for the apoptosis induction. Thus, to investigate whether the selected inhibitor induces cell death *via* apoptosis or not, we have performed Annexin-V/PI staining using flow cytometry. Cells were treated with IC_50_ concentration of each molecule for 48 h and processed for apoptosis analysis. The results showed that these compounds induce the apoptosis in MCF-7 and A549 cells (Fig. 7). The compound H4 induces apoptosis in 30.29% of MCF-7 cells and 28.5% of A549 cells, whereas compound H19 induces apoptosis in 26.71% of MCF-7 cells and 24.25% of A549 cells, respectively (Fig. 7A and B). Consistent to the cell proliferation, colonization and migration, these results suggest that compound H4 and H19 induces the apoptosis in MCF-7 and A549 cells.

## 4 Conclusions

In conclusion, the present study specifies that selected hydrazone derivatives effectively bind to the active site of MARK4 and thus potentially inhibit its activity. The cell-based assays further support the anticancer potential of compounds H4 and H19. Overall findings revealed that the present chemical scaffold may be further exploited in the design and development of MARK4-specific inhibitors with remarkable anticancer potential and minimal side effects.

## Funding

This work is supported by Department of Science and Technology, Government of India (Grant No: EMR/2015/002372).

## Conflicts of interest

Author declares no conflict of interest.

## Supplementary Material

RA-010-D0RA00453G-s001
