# Supplementary material for: Discovery of 4-(2-(dimethylamino)ethoxy)benzohydrazide derivatives as prospective microtubule affinity regulating kinase 4 inhibitors
Source: RSC Adv. 2020 May 27;10(34):20129–37. doi: 10.1039/d0ra00453g (PMC9054212; doi:10.1039/d0ra00453g)
Supplement: RA-010-D0RA00453G-s001 [file RA-010-D0RA00453G-s001.pdf]

Electronic Supplementary Material (ESI) for RSC Advances.
This journal is © The Royal Society of Chemistry 2020

Discovery of 4-(2-(dimethylamino)ethoxy)benzohydrazide derivatives as prospective microtubule affinity regulating kinase 4 inhibitor

Nashrah Sharif Khan[a], Parvez Khan[b], Afreen Inam[c], Kamal Ahmad[b], Mohd Yousuf[b], Asimul Islam[b], Sher Ali[b], Amir Azam[c], Mohammad Husain[a*] and Md. Imtaiyaz Hassan[b,*]

[1]Department of Biotechnology, [2]Centre for Interdisciplinary Research in Basic Sciences, [3]Department of Chemistry, Jamia Millia Islamia, Jamia Nagar, New Delhi, Delhi-110025, India

* *Corresponding authors*

E-mail address: mhussain2@jmi.ac.in (M. Hussain)

E-mail address: mihassan@jmi.ac.in (M. I. Hassan)

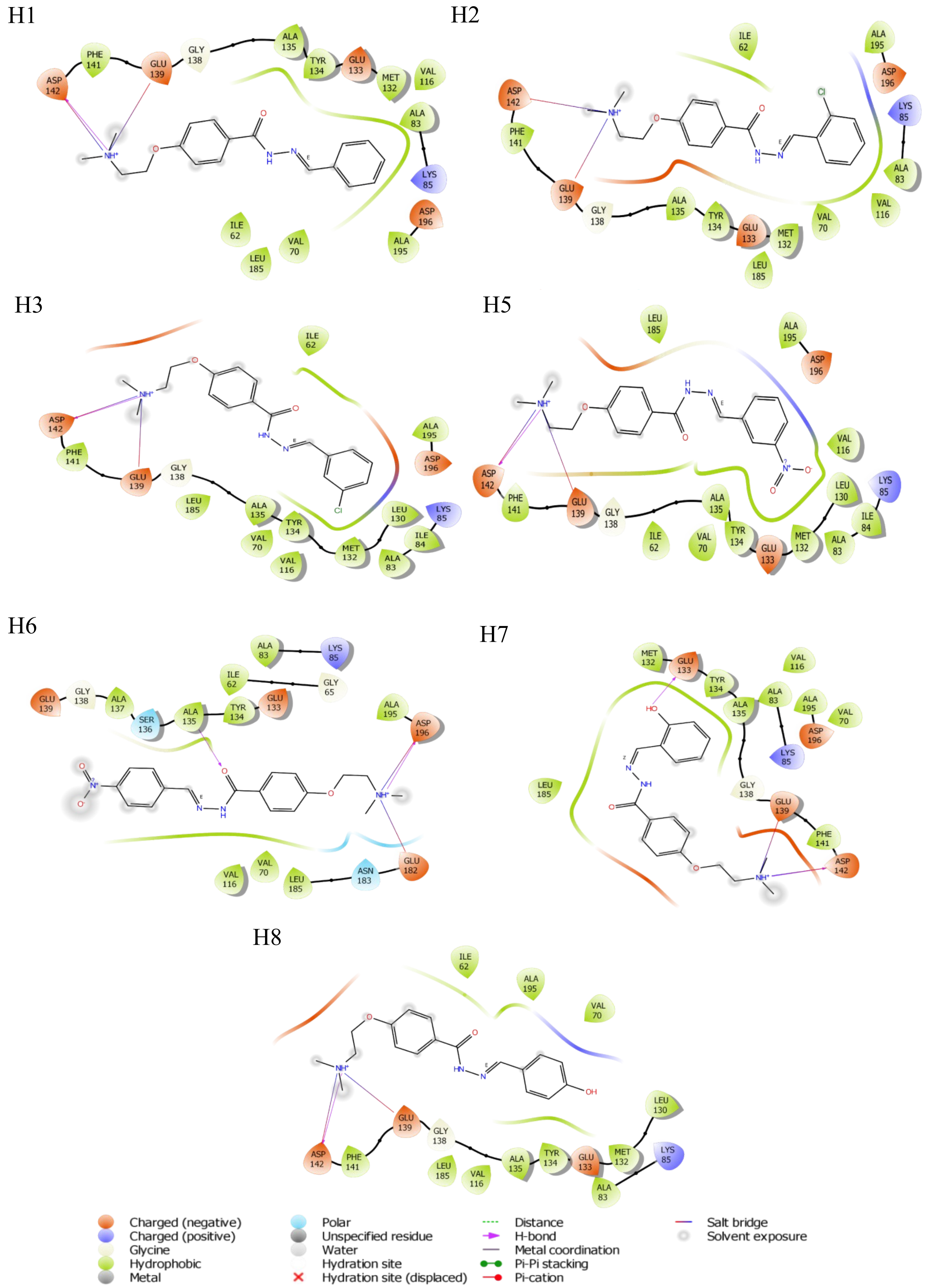

Figure S1: Molecular docking studies of selected compounds with MARK4: 2D schematic representation of the catalytic pocket residues of MARK4 with compound H1-H8.

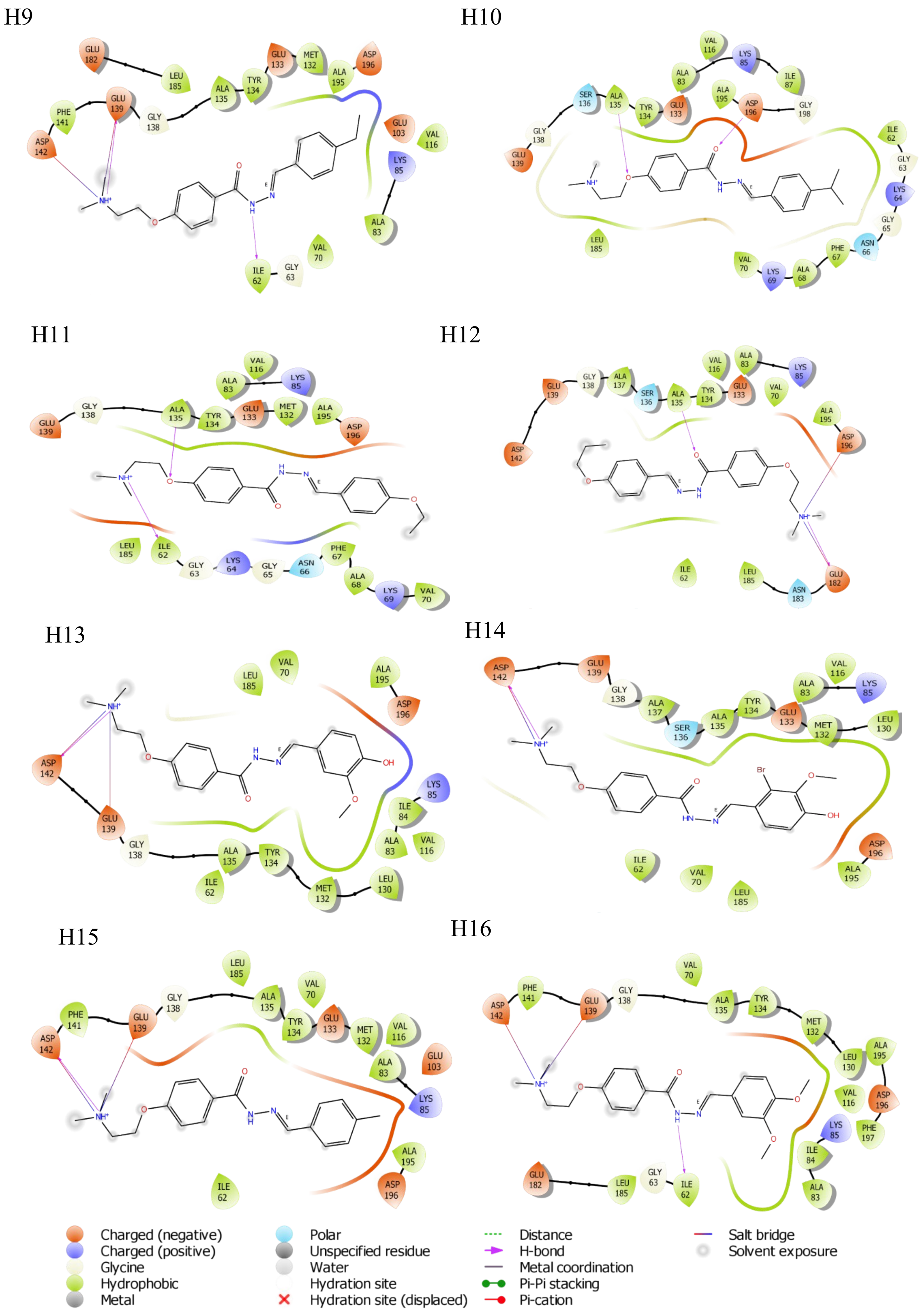

Figure S2: Molecular docking studies of selected compounds with MARK4: 2D schematic representation of the catalytic pocket residues of MARK4 with compound H9-H16.

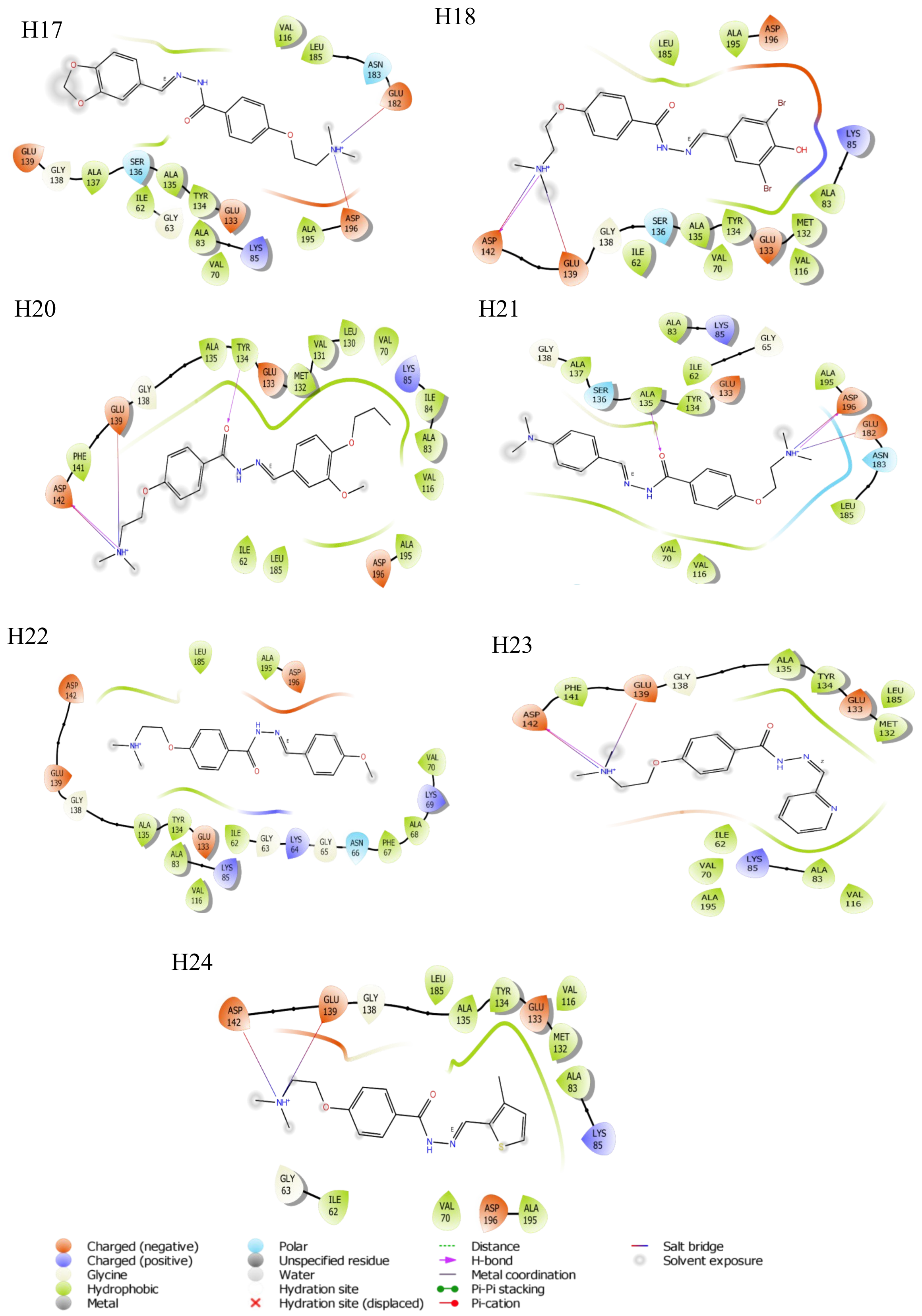

Figure S3: Molecular docking studies of selected compounds with MARK4: 2D schematic representation of the catalytic pocket residues of MARK4 with compound H17-H24.

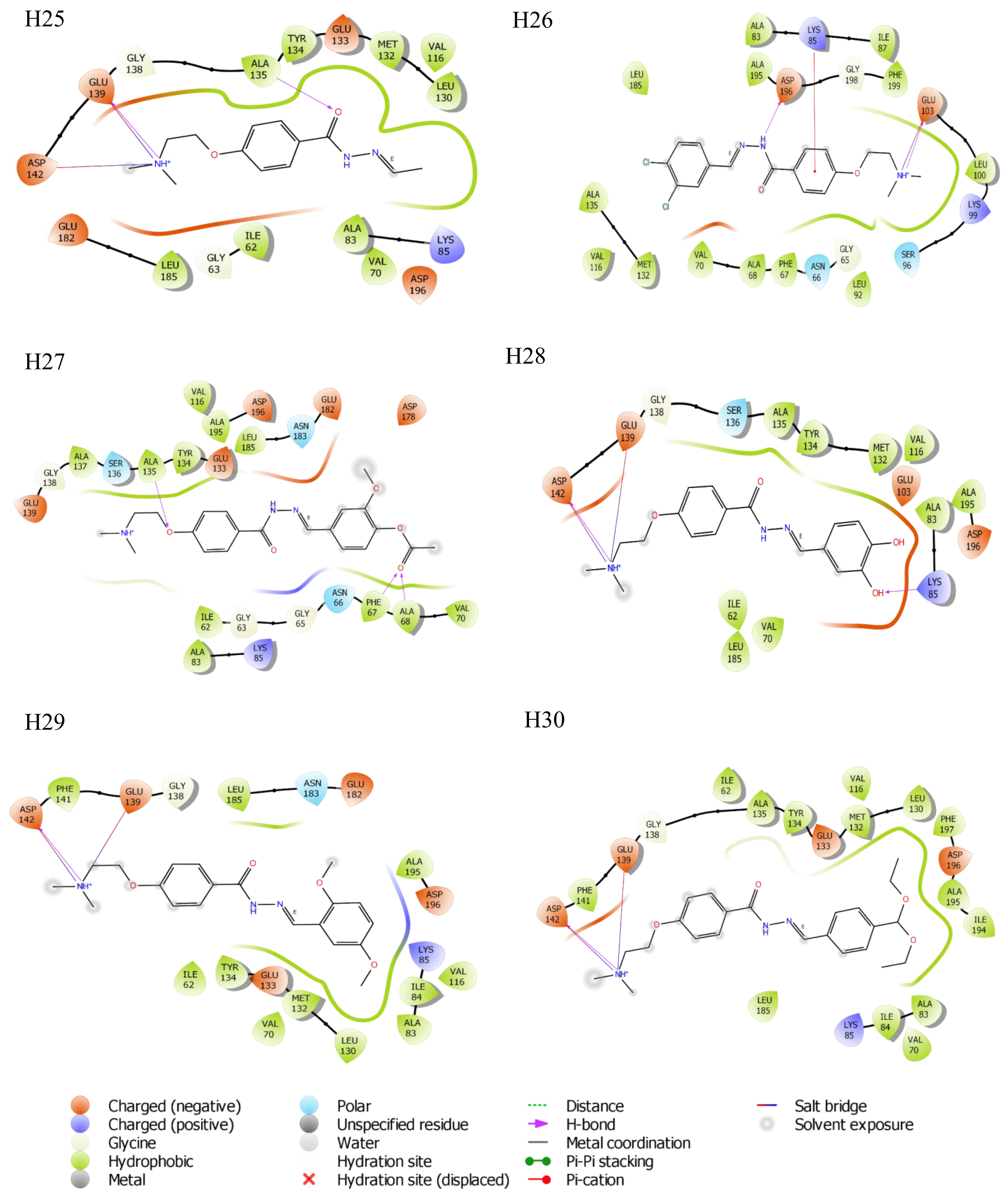

Figure S4: Molecular docking studies of selected compounds with MARK4: 2D schematic representation of the catalytic pocket residues of MARK4 with compound H25-H30.

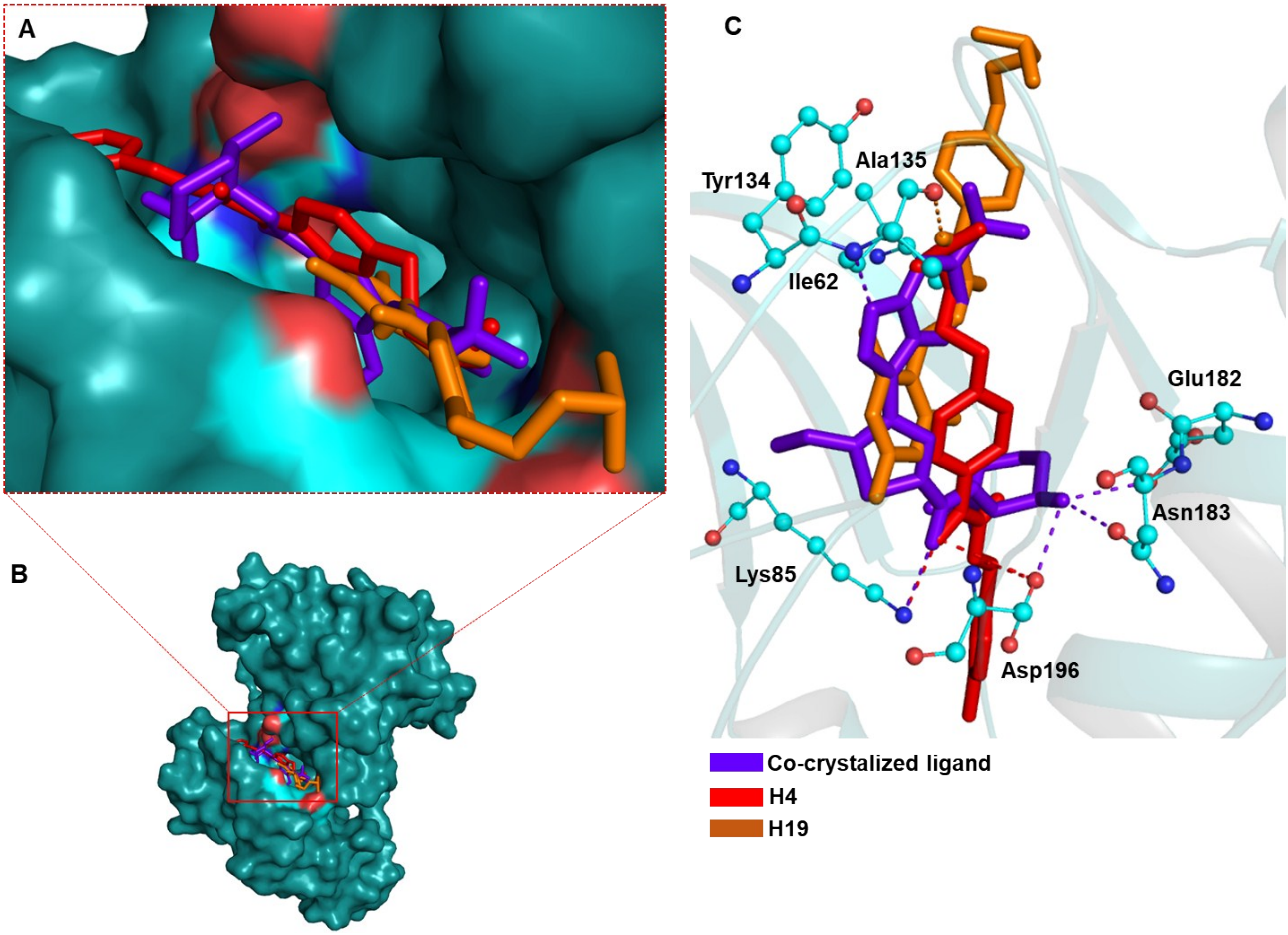

Figure S5: Molecular docking studies of selected compounds and co-crystalized/known inhibitor with MARK4: **(A)** 3D focused surface view representation of binding pocket with all three selected molecules. **(B)** 3D surface representation of docked complex of MARK4 with three selected molecules in the binding pocket. **(C)** The common catalytic pocket residues of MARK4 with compound H4, H19 and co-crystalized ligand.

Experimental Protocol

Materials and Methods

All the chemicals were purchased from Merck and Aldrich Chemical Company (USA). Precoated aluminum sheets (silica gel 60 F254, Merck Germany) were used for thin-layer chromatography (TLC) and were visualized under UV light. IR spectra were recorded on Bruker FT-IR spectrophotometer under neat condition on a Zn Se crystal. ^{1}H NMR was recorded on Bruker Spectrospin DPX 300 MHz using $CDCl_3$ as a solvent and tetramethylsilane (TMS) as an internal standard. Splitting patterns are designated as follows; s, singlet; d, doublet; m, multiplet. Chemical shift values are given in ppm. Electrospray (ES) mass spectra were carried out on Microtof-Q II 10262. Melting points were recorded on a Veego melting point apparatus (model REC-2203882) and were uncorrected. Elemental analyses were performed in an Elementar Vario analyzer and the results lay within ± 0.3% of the theoretical values.

Synthesis of Methyl-4-(2-(dimethylamino)ethoxy)benzoate (1): Acidic esterification of p-hydroxybenzoic acid with methanol yielded the ester, which was used for the synthesis of **(1)**. A solution of THF and potassium tert-butoxide was stirred along with addition of the ester *p*-hydroxymethylbenzoate, (32.89mmol) refluxed for 30 minutes followed by addition of 2-Dimethylaminoethylchloride (4.7384g, 32.89mmol) and was further refluxed for 50 h. The reaction mixture on extraction with ethyl acetate and water gave a viscous mass, which was the desired poduct. Brown viscous solid, Yield 68%, ^{1}H NMR ($CDCl_3$, 300MHz, δppm): 7.99(d, 2H, *J*=8.4,Ar-H), 6.94(d, 2H, *J*=8.7, Ar-H), 4.11(t, 2H, *J*=5.7Hz, -OCH_2), 3.88(ss, 3H, $COOCH_3$), 2.75(t, 2H, *J*=5.4Hz, -CH_2), 2.34 (ss, 6H, $N(CH3)_2$); IR *v*max (cm^{-1}): 2948(ArC-H, C-H str), 1712(COO ester group), 1604(C=O str), 1247(Ar-O-C,C-O-C asym. str), 846*(p*-disubstituted

benzene,C-H out of plane bending). Elemental analysis calcd (%) for $C_{12}H_{17}NO_3$: C 64.55, H 7.67, N 6.27; found: C 64.40, H 7.54, N 6.53.

Synthesis of *N*- substituted-4-(2-(dimethylamino)ethoxy)benzohydrazide (3-30): The mixture of hydrazine hydrate and methyl-4-(2-(dimethylamino)ethoxy)benzoate **(1)** was refluxed in ethanol for 6h to give 4-(2-(dimethylamino)ethoxy) benzohydrazide **(2)**. A mixture of hydrazide (2) and appropriate aromatic aldehydes in absolute ethanol (50ml) was refluxed for 4hrs with continuous stirring. On reaction completion (TLC) the cooled reaction mixture gave the solid product which was filtered and recrystallized in ethanol yielded the final product **(3-30) [1].**

***N'*-Benzylidene-4-(2-(dimethylamino)ethoxy)benzohydrazide (2):** Off white solid; ^{1}H NMR ($CDCl_3$, 300MHz, δppm): 7.72-7.69(m, 2H, Ar-H), 7.27(bs, 1H, NH), 6.96-6.93(m, 2H, Ar-H), 4.10 (t, 2H, *J*=5.7Hz, OCH_2), 2.74(t, 2H, *J*=5.4Hz,CH_2), 2.34 (ss, 6H, $N(CH3)_2$). IR *v*max (cm^{-1}): 3317($NH_{2,}$ N-H str) , 2973(ArC-H, C-H str), 1605(C=O str), 1250(Ar-O-C,C-O-C asym. str), 1172(N-N), 846(*p*-disubstituted benzene,C-H out of plane bending). Elemental analysis calcd (%) for $C_{11}H_{17}N_3O_2$: C 59.17, H 7.67, N 18.82; found: C 59.10, H 7.54, N 18.93.

***N'*-Benzylidene-4-(2-(dimethylamino)ethoxy)benzohydrazide (3):** White solid, Yield: 85%, m.pt. 172-176°c ; ^{1}HNMR ($CDCl_3$, 300MHz, δppm**):** 9.43 (bs,1H, -NH), 8.07(d, 2H, *J*=6.9Hz, Ar-H), 8.31 (s, 1H, -CHN),7.88-7.72 (m, 2H, Ar-H), 7.48-7.37 (m, 3H, Ar-H), 6.98 (d, 2H, *J*=8.7Hz, Ar-H), 4.12(t, 2H, *J*=5.7Hz, OCH_2), 2.77 (t, 2H, *J*=5.7Hz, CH_2), 2.36 (ss, 6H, $N(CH_3)_2$), 1.91 (s, 5H, Ar-H). IR v_{max} (cm^{-1}): 3363 ($NH_{2,}$ N-H str) , 3022 (ArC-H, C-H str), 1699 (C=O str), 1250 (Ar-O-C, C-O-C asym. str), 1171 (N-N); Elemental analysis calcd (%) for $C_{18}H_{21}N_3O_2$: C 69.43, H 6.80, N 13.49; found: C 69.14, H 6.54, N 13.33; ES-MS: m/z 312.52 (M+1).

***N'*-(2-chlorobenzylidene)-4-(2-(dimethylamino)ethoxy)benzohydrazide (H2):** Colourless solid, Yield: 85%, m.pt. 95-100°c; ^{1}H NMR ($CDCl_3$, 300MHz, δppm): 9.36 (bs,1H, -NH), 8.19 (s, 1H, CHN), 7.89(bs, 2H, Ar-H), 7.38-7.29(m, 4H, Ar-H), 7.01 (d, 2H, *J*=8.7, Ar-H), 4.13 (t, 2H, *J*=6Hz, OCH_2), 2.76 (t, 2H, *J*=5.7Hz, CH_2), 2.35 (ss, 6H, $N(CH_3)_2$). IRν_{max} (cm^{-1}): 3184 (N-H str) , 1649 (C=O str), 1554 (N=CH), 1256 (Ar-O-C, C-O-C asym. str), 1177(N-N); Elemental analysis calcd (%) for $C_{18}H_{20}N_3OCl$: C 62.52, H 5.83, N 12.15; found: C 62.34, H 5.54, N 12.33; ES-MS: m/z 346.16 (M+1).

***N'*-(3-chlorobenzylidene)-4-(2-(dimethylamino)ethoxy)benzohydrazide (H3):** Colourless solid, Yield: 82%, m.pt. 95-99°c; ^{1}H NMR ($CDCl_3$, 300MHz, δppm): 9.21 (bs,1H, -NH), 8.26(s, 1H, CHN), 7.87 (bs, 2H, Ar-H), 7.73 (bs, 1H, Ar-H),7.58 (bs, 1H, Ar-H), 7.37-7.29 (m, 2H, Ar-H), 7.00 (bs, 1H, CHN), 4.13 (t, 2H, *J*=5.7Hz, OCH_2), 2.76 (t, 2H, *J*=5.4Hz, CH2), 2.35(ss, 6H, $N(CH_3)_2$). IR ν_{max} (cm^{-1}): 3061(N-H str) , 3061(ArC-H, C-H str), 1643(C=O str), 1248(Ar-O-C, C-O-C asym. str), 1552(-N=CH), 1182(N-N); Elemental analysis calcd (%) for $C_{18}H_{20}N_3OCl$: C 62.52, H 5.83, N 12.15; found: C 62.34, H 5.54, N 12.33; ES-MS: m/z 346.15 (M+1).

***N'*-(4-chlorobenzylidene)-4-(2-(dimethylamino)ethoxy)benzohydrazide (H4):** Colourless solid, Yield: 84%, m.pt. 186-190°c; ^{1}H NMR ($CDCl_3$, 300MHz, δppm): 9.37(bs,1H, -NH), 8.28(s, 1H, CHN), 7.86(bs, 2H, Ar-H), 7.65 (bs, 2H, Ar-H),7.37 (d, 2H, *J*=8.1, Ar-H), 6.99 (d, 2H, *J*=8.4, Ar-H), 4.12 (t, 2H*J*=5.1Hz, OCH_2), 2.75(t, 2H, *J*=5.7Hz, CH_2), 2.35(ss, 6H, $N(CH_3)_2$). IR νmax (cm^{-1}): 3392 (N-H str) , 1695 (C=O str), 1565 (N=CH), 1257 (Ar-O-C, C-O-C asym. str), 1178 (N-N); Elemental analysis calcd (%) for $C_{18}H_{20}N_3OCl$: C 62.52, H 5.83, N 12.15; found: C 62.34, H 5.54, N 12.33; ES-MS: m/z 346.28 (M+1).

***N'*-(3-Nitrobenzylidene)-4-(2-(dimethylamino)ethoxy)benzohydrazide (H5):** Colourless solid, Yield: 82%, m.pt. 125-130°c ; ^{1}H NMR ($CDCl_3$, 300MHz, δppm): 9.43(bs,1H, -NH), 8.46(s, 1H, CHN), 8.24(d, 2H,*J*=8.7, Ar-H), 8.13(bs, 1H, Ar-H) 7.89(d, 2H,*J*=7.5, Ar-H), 7.58(t, 1H, *J*=8.1, Ar-H), 7.01 (d, 2H, *J*=8.7, Ar-H), 4.13(t, 2H, *J*=5.7Hz, OCH_2), 2.35 (ss, 6H, $N(CH_3)_2$). IR *v*max (cm^{-1}): 3204(N-H str) , 1684(C=O str), 1562(N=CH), 1259(Ar-O-C, C-O-C asym. str), 1177(N-N); Elemental analysis calcd (%) for $C_{18}H_{20}N_4O_4$: C 60.66, H 5.66, N 15.72; found: C 60.34, H 5.54, N 15.43; ES-MS: m/z 357.11 (M+1).

***N'*-(4-Nitrobenzylidene)-4-(2-(dimethylamino)ethoxy)benzohydrazide (H6):** Yellow solid, Yield: 82%, m.pt.96-100°C ; ^{1}H NMR ($CDCl_3$, 300MHz, δppm): 9.84(bs,1H, -NH), 8.46(s, 1H, CHN), 8.25(d, 2H,*J*=8.7, Ar-H), 7.87 (d, 4H,*J*=6.9, Ar-H) 6.98(d, 2H,*J*=8.7, Ar-H), 4.19(t, 2H, *J*=5.4Hz, OCH_2), 2.87(t,2H,*J*=5.7Hz, CH_2), 2.42 (ss, 6H, $N(CH_3)_2$). IR *v*max (cm^{-1}): 3367 (N-H str) , 1653(C=O str), 1565(N=CH), 1247 (Ar-O-C, C-O-C asym. str), 1168(N-N); Elemental analysis calcd (%) for $C_{18}H_{20}N_4O_4$: C 60.66, H 5.66, N 15.72; found: C 60.34, H 5.54, N 15.43; ES-MS: m/z 357.32 (M+1).

***N'*-(2-hydroxybenzylidene)-4-(2-(dimethylamino)ethoxy)benzohydrazide (H7):** Yellow solid, Yield: 70%, m.pt. 149-153°C ; ^{1}H NMR ($CDCl_3$, 300MHz, δppm): 11.06 (bs, 1H), 9.12 (s,1H), 8.46 (s, 1H), 7.82 (d, 2H,*J*=8.7), 7.33-7.33 (m, 1H), 7.23-7.20 (m, 1H), 7.00-6.97 (m, 3H), 4.13 (t, 2H, *J*=5.4Hz), 2.77(t,2H,*J*=5.7Hz), 2.35 (ss, 6H). IR *v*max (cm^{-1}): 3355 (N-H str) , 1646(C=O str), 1561(N=CH), 1252 (Ar-O-C, C-O-C asym. str), 1150(N-N); Elemental analysis calcd (%) for $C_{18}H_{21}N_3O_3$: C 66.04, H 6.47, N 12.84; found: C 66.14, H 6.47, N 12.80 ES-MS: m/z 328.00 (M+1).

***N'*-(4-hydroxybenzylidene)-4-(2-(dimethylamino)ethoxy)benzohydrazide (H8):** Yellow solid, Yield: 62%, m.pt.109-114°C ; ^{1}H NMR ($CDCl_3$, 300MHz, δppm): 11.51 (s,1H, -NH), 9.92

(s, 1H, OH), 8.33(s, 1H, CHN), 7.88(d, 2H, *J*=6.3Hz, Ar-H), 7.55(d, 2H,*J*=8.7Hz, Ar-H), 7.06 (d, 2H, *J*=6.3Hz, Ar-H), 6.84 (d, 2H, *J*=6.0 Hz, Ar-H), 4.13 (t, 2H, *J*=5.4Hz, OCH_2), 2.81 (t, 2H, *J*=5.7Hz, CH_2), 2.22 (ss, 6H, $N(CH_3)_2$); IR*v*max (cm^{-1}): 3301 (N-H str) , 1644 (C=O str), 1557 (N=CH), 1283 (Ar-O-C, C-O-C asym. str), 1166 (N-N);. Elemental analysis calcd (%) for $C_{18}H_{21}N_3O_3$: C 66.04, H 6.47, N 12.84; found: C 66.34, H 6.45, N 12.83; ES-MS: m/z 328.55 (M+1).

***N'*-(4-ethylbenzylidene)-4-(2-(dimethylamino)ethoxy)benzohydrazide (H9):** solid, Yield: 60%, m.pt.153-157°C ; ^{1}H NMR ($CDCl_3$, 300MHz, δppm): 9.11 (bs,1H), 8.24 (s, 1H), 7.85 (bs, 2H), 7.65 (bs, 2H),7.26-7.21 (m, 2H), 6.99 (d, 2H, *J*=7.2Hz), 4.14(t, 2H, *J*=5.7Hz), 2.77 (t, 2H, *J*=5.4Hz), 1.27 (ss, 6H). IR*v*max (cm^{-1}): 3357 (N-H str) , 1648 (C=O str), 1560 (N=CH), 1270 (Ar-O-C, C-O-C asym. str), 1173 (N-N); Elemental analysis calcd (%) for $C_{20}H_{25}N_3O_2$: C 70.77, H 7.42, N 12.38; found: C 70.64, H 7.54, N 12.43; ES-MS: m/z 340.10 (M+1).

***N'*-(4-isopropylbenzylidene)-4-(2-(dimethylamino)ethoxy)benzohydrazide (H10):** solid, Yield: 60%, m.pt.148-153°C; ^{1}H NMR ($CDCl_3$, 300MHz, δppm): 9.41 (bs,1H), 8.24 (s, 1H), 7.96-7.84 (m, 2H), 7.68-7.61 (m, 2H), 7.25-7.24 (m, 2H), 6.99 (d, 2H, *J*=8.7Hz), 4.14 (t, 2H, *J*=5.4Hz), 2.97-2.91 (m, 1H), 2.78 (t, 2H, *J*=5.7Hz), 2.35 (s, 6H), 1.27 (ss, 6H); IR*v*max (cm^{-1}): 3384 (N-H str) , 1647 (C=O str), 1557 (N=CH), 1250 (Ar-O-C, C-O-C asym. str), 1176 (N-N); Elemental analysis calcd (%) for $C_{21}H_{27}N_3O_2$: C 71.36, H 7.70, N 11.89; found: C 71.35, H 7.70, N 11.88; ES-MS: m/z 354.25 (M+1).

***N'*-(4-ethoxybenzylidene)-4-(2-(dimethylamino)ethoxy)benzohydrazide (H11):** solid, Yield: 65%, m.pt.162-165°C ; ^{1}H NMR ($CDCl_3$, 300MHz, δppm): 9.24 (bs,1H), 8.21 (s, 1H), 7.84 (bs, 2H), 7.67 (bs, 2H), 6.98 (d, 2H, *J*=8.7Hz), 6.90 (d, 2H, J=8.7Hz), 4.13 (t, 2H, *J*=5.4Hz), 4.07-4.02 (m, 2H), 2.77 (t, 2H, *J*=5.7Hz), 2.34 (s, 6H), 1.44(t, 3H, J=6.9Hz); IR*v*max (cm^{-1}): 3301 (N-

H str) , 1644 (C=O str), 1557 (N=CH), 1250 (Ar-O-C, C-O-C asym. str), 1167 (N-N); Elemental analysis calcd (%) for $C_{20}H_{25}N_3O_3$: C 67.58, H 7.09, N 11.82; found: C 67.58, H 7.09, N 11.85; ES-MS: m/z 356.10 (M+1).

***N'*-(4-propoxybenzylidene)-4-(2-(dimethylamino)ethoxy)benzohydrazide (H12):** solid, Yield: 72%, m.pt.189-193°C; ^{1}H NMR ($CDCl_3$, 300MHz, δppm): 9.08 (bs,1H), 8.19 (s, 1H), 7.96-7.83 (m, 2H), 7.65-7.60 (m, 2H), 6.99 (d, 2H, *J*=8.7Hz), 6.90 (d, 2H, J=9.0Hz), 4.65 (m, 1H), 4.14 (t, 2H, *J*=5.4Hz), 2.77 (t, 2H, *J*=5.7Hz), 2.34 (s, 6H), 1.36(d, 6H, J=6.0Hz); IR*ν*max (cm^{-1}): 3361 (N-H str) , 1644 (C=O str), 1563 (N=CH), 1240 (Ar-O-C, C-O-C asym. str), 1142 (N-N); Elemental analysis calcd (%) for $C_{21}H_{27}N_3O_3$: C 68.27, H 7.37, N 11.37; found: C 68.27, H 7.39, N 11.34; ES-MS: m/z 370.22 (M+1).

***N'*-(4-hydroxy-3-methoxybenzylidene)-4-(2-(dimethylamino)ethoxy)benzohydrazide (H13):** solid, Yield: 76%, m.pt.145-149°C; ^{1}H NMR ($CDCl_3$, 300MHz, δppm): 11.02 (s, 1H), 8.24 (s, 2H), 7.91-7.49 (m, 3H), 7.02-6.87 (m, 4H), 4.13 (t, 2H, *J*=5.4Hz), 3.94 (s, 3H) 2.78-2.74 (m, 2H), 2.33 (s, 6H); IR*ν*max (cm^{-1}): 3358 (N-H str) , 1640 (C=O str), 1504 (N=CH), 1241 (Ar-O-C, C-O-C asym. str), 1173 (N-N); Elemental analysis calcd (%) for $C_{19}H_{23}N_3O_4$: C 63.85, H 6.49, N 11.76; found: C 63.89, H 6.52, N 11.75; ES-MS: m/z 358.11(M+1).

***N'*-(2-bromo-4-hydroxy-3-methoxybenzylidene)-4-(2-(dimethylamino)ethoxy) benzohydrazide (H14):** solid, Yield: 68%, m.pt.195-199°C; ^{1}H NMR ($CDCl_3$, 300MHz, δppm): 11.68 (s,1H), 10.12 (bs, 1H), 8.29 (s, 1H), 7.89 (d, 2H, *J*=5.7Hz), 7.40-7.30 (m, 2H), 7.10-7.07 (m, 2H), 4.14 (t, 2H, *J*=5.4Hz), 3.89 (s, 3H), 2.64 (t, 2H, *J*=5.7Hz), 2.22 (s, 6H); IR*ν*max (cm^{-1}): 3356 (N-H str) , 1640 (C=O str), 1502 (N=CH), 1250 (Ar-O-C, C-O-C asym. str), 1169 (N-N); Elemental analysis calcd (%) for $C_{19}H_{22}BrN_3O_4$: C 52.30, H 5.08, N 9.63; found: C 52.33, H 5.18, N 9.66; ES-MS: m/z 437.36 (M+1).

***N'*-(4-methylbenzylidene)-4-(2-(dimethylamino)ethoxy)benzohydrazide (H15):** solid, Yield: 55%, m.pt.162-166°C; ^1H NMR ($CDCl_3$, 300MHz, δppm): 9.11 (bs,1H), 8.22 (s, 1H), 7.83 (bs, 2H), 7.65 (bs, 2H), 7.22 (d, 2H, J=8.1Hz), 7.00(d, 2H, *J*=8.1Hz), 4.14(t, 2H, *J*=5.4Hz), 2.78 (t, 2H, *J*=5.7Hz), 2.38 (ss, 9H); IR*ν*max (cm^{-1}): 3353 (N-H str) , 1647 (C=O str), 1562(N=CH), 1276 (Ar-O-C, C-O-C asym. str), 1176 (N-N); Elemental analysis calcd (%) for $C_{19}H_{23}N_3O_2$: C 70.13, H 7.12, N 12.91; found: C 70.15, H 7.16, N 12.89; ES-MS: m/z 326.41 (M+1).

***N'*-(3,4-dimethoxybenzylidene)-4-(2-(dimethylamino)ethoxy)benzohydrazide (H16):** solid, Yield: 64%, m.pt.124-127°C; ^1H NMR ($CDCl_3$, 300MHz, δppm): 9.43 (bs,1H), 8.22 (s, 1H), 7.85 (bs, 2H), 7.48 (bs, 2H), 7.10 (d, 1H, J=8.4Hz), 6.97(d, 2H, *J*=8.4Hz), 6.85 (d, 1H, J=8.1Hz), 4.12 (t, 2H, *J*=5.4Hz), 3.91 (ss, 6H), 2.77 (t, 2H, *J*=5.4Hz), 2.34 (s, 6H); IR*ν*max (cm^{-1}): 3354 (N-H str), 1641 (C=O str), 1540 (N=CH), 1240 (Ar-O-C, C-O-C asym. str), 1165 (N-N); Elemental analysis calcd (%) for $C_{20}H_{25}N_3O_4$: C 64.67, H 6.78, N 11.31; found: C 64.65, H 6.79, N 11.32; ES-MS: m/z 372.10 (M+1).

***N'*-(benzo[1,3]dioxol-5-ylmehylene)-4-(2-(dimethylamino)ethoxy)benzohydrazide (H17):** solid, Yield: 69%, m.pt.146-149°C; ^1H NMR ($CDCl_3$, 300MHz, δppm): 9.21(bs,1H), 8.20 (s, 1H), 7.84 (bs, 2H), 7.39 (bs, 1H), 7.06 (d, 1H, J=8.1Hz), 6.98 (d, 2H, *J*=8.7Hz), 6.81 (d 1H, J=8.1Hz), 6.00 (ss, 2H), 4.13(t, 2H, *J*=5.4Hz), 2.77 (t, 2H, *J*=5.7Hz), 2.34 (s, 6H); IR*ν*max (cm^{-1}): 3299 (N-H str) , 1641 (C=O str), 1561 (N=CH), 1250 (Ar-O-C, C-O-C asym. str), 1171 (N-N); Elemental analysis calcd (%) for $C_{19}H_{21}N_3O_4$: C 64.21, H 5.96, N 11.82; found: C 64.20, H 5.97, N 11.80; ES-MS: m/z 356.10 (M+1).

***N'*-(3,5-dibromo-4-nydroxybenzylidene)-4-(2-(dimethylamino)ethoxy)benzohydrazide (H18):** solid, Yield: 56%, m.pt.148-152°C; ^1H NMR ($CDCl_3$, 300MHz, δppm): 11.63 (s,1H), 8.19(s, 1H), 7.89-7.87 (m, 2H), 7.77-7.61(m, 2H), 7.06-7.04 (m, 2H), 4.27 (t, 2H, *J*=5.4Hz), 2.84

(t, 2H, *J*=5.7Hz), 2.43 (s, 6H); IR*ν*max (cm^{-1}): 3356 (N-H str) , 1612 (C=O str), 1571(N=CH), 1252 (Ar-O-C, C-O-C asym. str), 1175 (N-N); Elemental analysis calcd (%) for $C_{18}H_{19}Br_2N_3O_3$: C 44.56, H 3.95, N 8.66; found: C 44.56, H 3.95, N 8.66; ES-MS: m/z 486.05 (M+1).

***N'*-(2,4,6-trimethylbenzylidene)-4-(2-(dimethylamino)ethoxy)benzohydrazide (H19):** Solid, Yield: 60%, m.pt.193-196°C; 1H NMR (CDCl3, 400MHz, δppm): 11.57 (s,1H, NH), 8.73 (s, 1H, CHN), 7.90-7.89 (m, 2H, Ar-H), 7.07-7.05 (m, 2H, Ar-H), 6.91 (s, 2H, Ar-H), 4.12 (t, 2H, J=5.4Hz, OCH2), 2.65(t, 2H, J=5.7Hz, CH2), 2.42 (s, 6H, CH3), 2.24(s, 3H, CH3), 2.21 (s, 6H, N(CH3)2); IR*ν*max (cm^{-1}): 3227 (N-H str) , 1643 (C=O str), 1542 (N=CH), 1255 (Ar-O-C, C-O-C asym. str), 1179 (N-N); Elemental analysis calcd (%) for $C_{21}H_{27}N_3O_2$: C 71.36, H 7.70, N 11.89; found: C 71.35, H 7.73, N 11.86; ES-MS: m/z 354.16 (M+1).

***N'*-(4-(dimethylamino)benzylidene)-4-(2-(dimethylamino)ethoxy)benzohydrazide (H21):** solid, Yield: 56%, m.pt.215-219°C; ^{1}H NMR ($CDCl_3$, 300MHz, δppm): 8.94(bs,1H), 8.10 (s, 1H), 7.91 (bs, 2H), 7.63 (bs, 2H), 6.98 (d, 2H, *J*=8.7Hz), 6.70 (d, 2H, *J*=8.7Hz), 4.31 (t, 2H, *J*=5.4Hz), 3.02 (ss, 6H), 2.79 (t, 2H, *J*=5.7Hz), 2.37 (s, 6H); IR*ν*max (cm^{-1}): 3352 (N-H str) , 1644 (C=O str), 1517 (N=CH), 1253 (Ar-O-C, C-O-C asym. str), 1177 (N-N); Elemental analysis calcd (%) for $C_{20}H_{26}N_4O_2$: C 67.77, H 7.39, N 15.81; found: C 67.75, H 7.40, N 15.81; ES-MS: m/z 355.16 (M+1).

4-(2-(dimethylamino)ethoxy)-*N*-(pyridine-2-ylmethylene)benzohydrazide (H23): solid, Yield: 52%, m.pt.98-102°C; ^{1}H NMR ($CDCl_3$, 300MHz, δppm): 9.38(bs,1H), 8.61 (s, 1H), 8.23-8.05 (m, 2H), 8.00-7.90 (m, 2H), 7.76 (t, 1H, J=7.8Hz), 7.31-7.28 (m, 1H), 7.013 (d, 2H, J=8.7Hz), 4.152 (t, 2H, *J*=5.4Hz), 2.781 (t, 2H, *J*=5.7Hz), 2.35 (s, 6H); IR*ν*max (cm^{-1}): 3379 (N-H str) , 1649 (C=O str), 1560 (N=CH), 1272 (Ar-O-C, C-O-C asym. str), 1179 (N-N); Elemental

analysis calcd (%) for $C_{17}H_{20}N_4O_2$: C 65.37, H 6.45, N 17.94; found: C 65.36, H 6.46, N 17.96; ES-MS: m/z 313.23(M+1).

4-(2-(dimethylamino)ethoxy)-N-((3-methylthiophene—2-yl)methylenene)benzohydrazide (H24): solid, Yield: 61%, m.pt.139-143°C; ^{1}H NMR ($CDCl_3$, 300MHz, δppm): 9.31 (bs,1H), 9.20 (s, 1H), 7.83 (bs, 2H), 7.27 (bs, 1H), 6.97 (d, 2H, *J*=8.7Hz), 6.84 (d, 2H, J=5.1Hz), 4.13 (t, 2H, *J*=5.4Hz), 2.77 (t, 2H, *J*=5.7Hz), 2.34 (s, 6H); IR*v*max (cm^{-1}): 3324 (N-H str) , 1642 (C=O str), 1558 (N=CH), 1244 (Ar-O-C, C-O-C asym. str), 1177 (N-N); Elemental analysis calcd (%) for $C_{17}H_{21}N_3O_2S$: C 61.61, H 6.39, N 12.68; found: C 61.61, H 6.39, N 12.65; ES-MS: m/z 332.49 (M+1).

Ferrocenyl-4-(2-(dimethylamino)ethoxy)benzohydrazide (H25): solid, Yield: 60%, m.pt.189-193°C; ^{1}H NMR ($CDCl_3$, 300MHz, δppm): 8.84 (bs,1H), 8.22 (bs, 1H), 7.81 (bs, 2H), 6.99 (d, 2H, *J*=8.7Hz),4.69 (bs, 2H), 4.41 (bs, 2H), 4.221 (s, 4H), 4.15 (t, 2H, *J*=5.4Hz), 2.78(t, 2H, *J*=5.7Hz), 2.36 (s, 6H); IR*v*max (cm^{-1}): 3397 (N-H str) , 1641 (C=O str), 1558 (N=CH), 1256 (Ar-O-C, C-O-C asym. str), 1188 (N-N); Elemental analysis calcd (%) for $C_{22}H_{25}FeN_3O_2$: C 62.96, H 5.39, N 10.01; found: C 62.95, H 5.39, N 10.04; ES-MS: m/z 421.50 (M+1).

***N’*-(3,4-dichlorobenzylidene)-4-(2-(dimethylamino)ethoxy)benzohydrazide (H26):** solid, Yield: 69%, m.pt.92-96°C; ^{1}H NMR ($CDCl_3$, 300MHz, δppm): 8.34 (bs,1H), 7.84(s, 1H), 7.81 (bs, 3H), 7.54 (bs, 1H), 7.47 (d, 1H, J=8.1Hz), 6.98 (d, 2H, *J*=8.4Hz), 4.14(t, 2H, *J*=5.4Hz), 2.74 (t, 2H, *J*=5.7Hz), 2.35 (s, 6H); IR*v*max (cm^{-1}): 3260 (N-H str) , 1649 (C=O str), 1554 (N=CH), 1247 (Ar-O-C, C-O-C asym. str), 1173 (N-N); Elemental analysis calcd (%) for $C_{18}H_{19}Cl_2N_3O_2$: C 56.85, H 5.04, N 11.05; found: C 56.85, H 5.02, N 11.02; ES-MS: m/z 381.28 (M+1).

2-methoxy-4-[2-{4-(dimethylamino)ethoxybenzoyl)hydrazinylidene}methyl]phenyl acetate (H27): solid, Yield: 56%, m.pt.78-83°C; ^{1}H NMR ($CDCl_3$, 300MHz, δppm): 9.66 (bs,1H), 8.10

(bs, 1H), 7.87(bs, 2H), 7.47 (s, 1H), 6.99-6.93 (m, 4H), 4.13 (t, 2H, *J*=6.0Hz), 3.82 (s, 3H), 2.78-2.75 (m, 2H), 2.35 (s, 6H), 2.31 (s, 3H); IR*v*max (cm^{-1}): 3329 (N-H str), 1646 (C=O str), 1554 (N=CH), 1284 (Ar-O-C, C-O-C asym. str), 1171 (N-N); Elemental analysis calcd (%) for $C_{21}H_{25}N_3O_5$: C 63.14, H 6.31, N 10.52; found: C 63.14, H 6.33, N 10.55; ES-MS: m/z 400.07(M+1).

***N'*-(3,4-dihydroxybenzylidene)-4-(2-(dimethylamino)ethoxy)benzohydrazide (H28):** solid, Yield: 64%, m.pt.191-195°C; ^{1}H NMR ($CDCl_3$, 300MHz, δppm): 11.47 (s,1H), 9.73-9.29 (bs, 2H, OH), 8.24 (s, 1H), 7.78-7.66 (m, 2H), 7.30 (s, 1H), 7.05-7.03 (m, 2H), 6.78-6.76 (m, 1H), 6.60-6.56 (m, 1H), 4.13 (t, 2H, *J*=5.4Hz), 2.63 (t, 2H, *J*=5.7Hz), 2.22(s, 6H) IR*v*max (cm^{-1}): 3314 (N-H str) , 1645 (C=O str), 1505 (N=CH), 1248 (Ar-O-C, C-O-C asym. str), 1176 (N-N); Elemental analysis calcd (%) for $C_{18}H_{21}N_3O_4$: C 62.96, H 6.16, N 12.24; found: C 62.93, H 6.17, N 12.25; ES-MS: m/z 344.64 (M+1).

***N'*-(2,5-dimethoxybenzylidene)-4-(2-(dimethylamino)ethoxy)benzohydrazide (H29):** solid, Yield: 59%, m.pt.121-124°C; ^{1}H NMR ($CDCl_3$, 300MHz, δppm): 9.22 (bs,1H), 8.57 (bs, 1H), 7.95 (bs, 2H), 7.64 (bs, 1H), 6.99-6.93 (m, 3H), 6.86-6.83 (m, 1H), 4.14- (t, 2H, J=4.8Hz), 3.82 (ss, 6H), 2.78 (t, 2H, *J*=4.5Hz), 2.36 (s, 6H); IR*v*max (cm^{-1}): 3336 (N-H str) , 1642 (C=O str), 1563 (N=CH), 1246 (Ar-O-C, C-O-C asym. str), 1170 (N-N); Elemental analysis calcd (%) for $C_{20}H_{25}N_3O_4$: C 64.67, H 6.78, N 11.31; found: C 64.67, H 6.78, N 11.31; ES-MS: m/z 372.18 (M+1).

***N'*-(4-(diethoxymethyl)benzylidene)-4-(2-(dimethylamino)ethoxy)benzohydrazide (H30):** solid, Yield: 69%, m.pt.70-74°C; ^{1}H NMR ($CDCl_3$, 300MHz, δppm): 9.05 (bs,1H), 7.86 (s, 1H), 7.72 (bs, 2H), 7.52 (d, 2H, J=8.1Hz), 7.00(d, 2H, *J*=8.7Hz), 5.52 (ss, 1H), 4.15 (t, 2H, *J*=5.7Hz), 3.62-3.49 (m, 4H), 2.79 (t, 2H, *J*=5.7Hz), 2.36 (s, 6H), 1.26 (t, 6H, J=7.2Hz); IR*v*max (cm^{-1}):

3389 (N-H str) , 1649 (C=O str), 1558 (N=CH), 1259 (Ar-O-C, C-O-C asym. str), 1176 (N-N); Elemental analysis calcd (%) for $C_{23}H_{31}N_3O_4$: C 66.81, H 7.56, N 10.16; found C 66.85, H 7.55, N 10.14; ES-MS: m/z 414.11 (M+1).

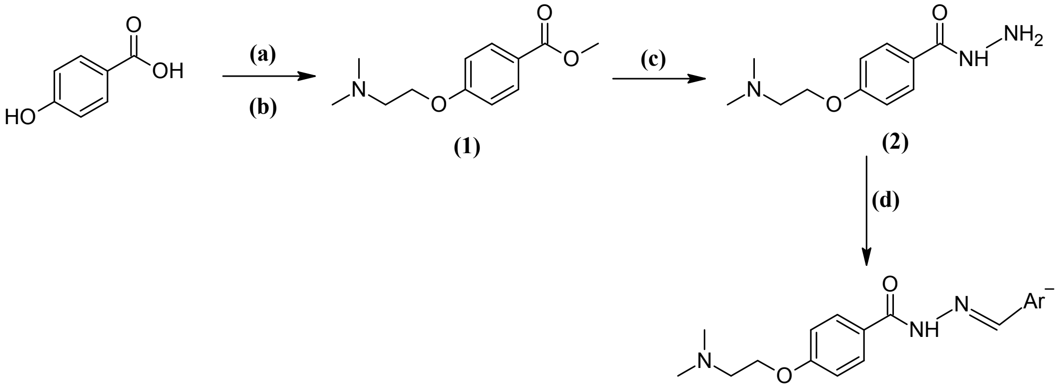

(3-30)

Scheme: *Synthesis of N-substituted -4-(2-(dimethylamino)ethoxy)benzohydrazides (3-30);* ***Reagents and conditions:*** *(a) MeOH, Few drops of sulfuric acid (b) THF, Potassium tert. Butoxide, 2-Dimethylaminoethylchloride (c) Hydrazine Hydrate, Ethanol (d) Different Substituted aldehydes, Ethanol.*

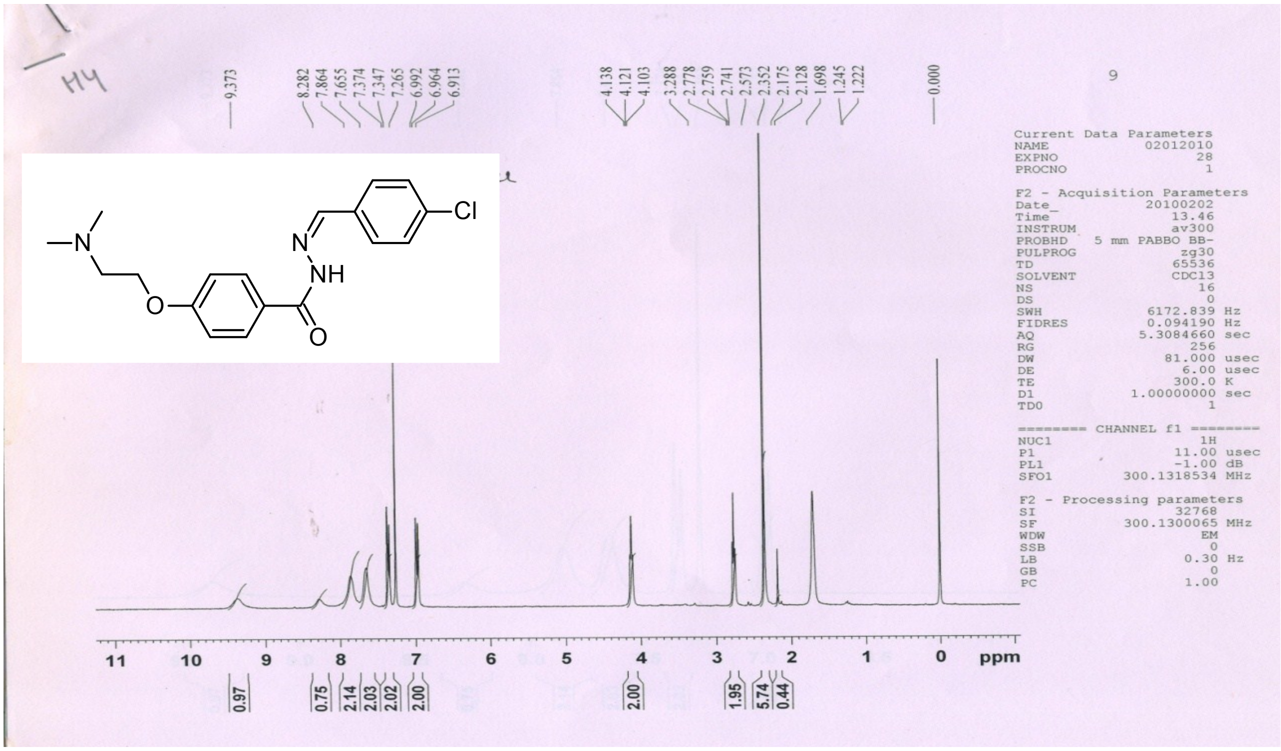

Figure S6: ^{1}H NMR spectra of compound H4.

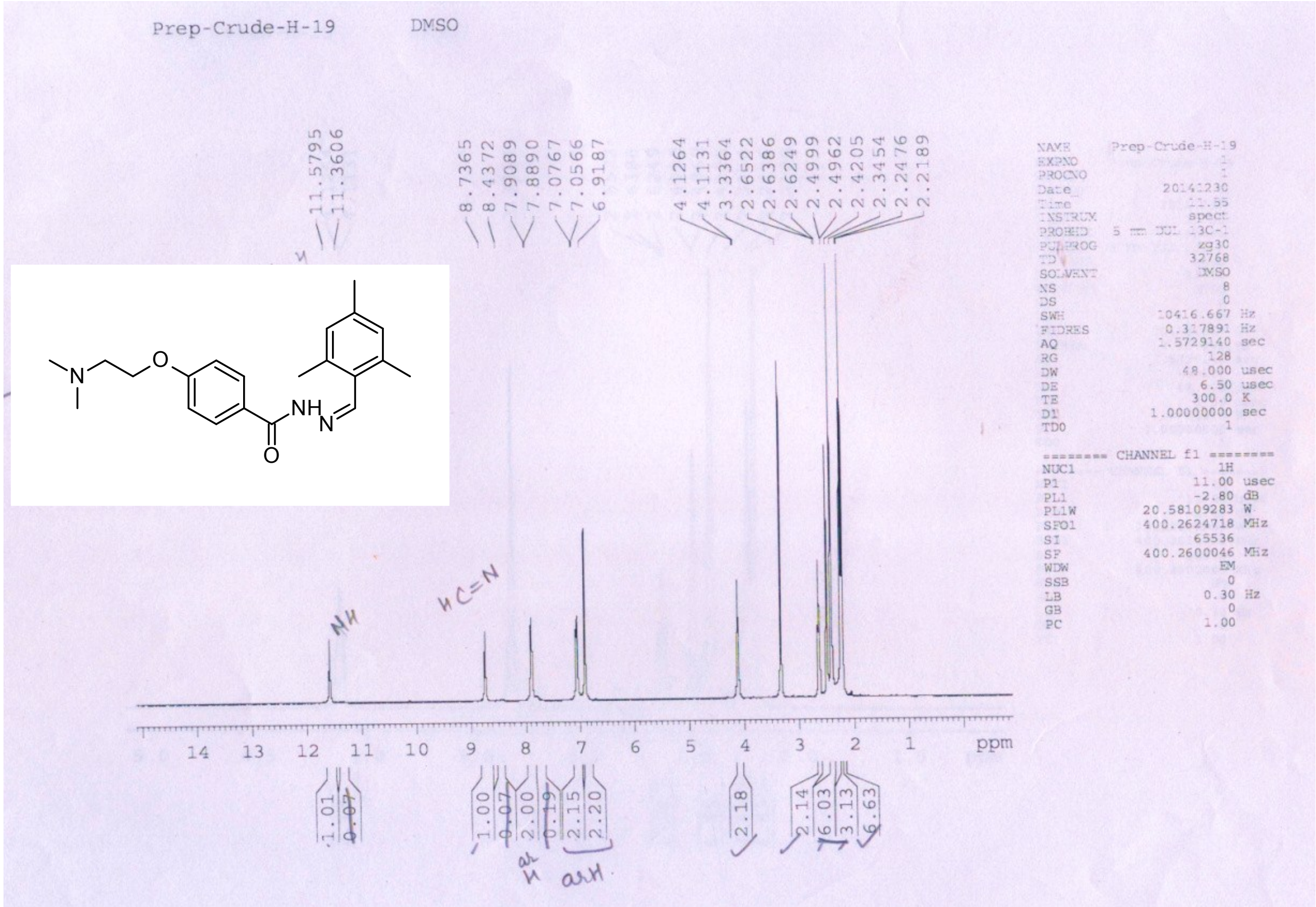

Figure S7: ^{1}H NMR spectra of compound H19

Refrence

1. A. Inam, S. Mittal, M.S. Rajala, F. Avecilla, A. Azam, Synthesis and biological evaluation of 4-(2-(dimethylamino)ethoxy)benzohydrazide derivatives as inhibitors of Entamoeba histolyica, Eur J Med Chem 124 (2016) 445-455.
